# The Drunkards’ Home

**Published:** 1871-04

**Authors:** 


### THE DRUNKARDS' HOME.

Whether drunkenness is a disease or an inappeasable appetite, the unfortunate individual ought to have our sympathy, and all the aid which can be given to save from the ruin which threatens. There is an "Inebriate Asylum" at Binghampton, New York, under the care of Dr. Day, the founder of the Washingtonian Home of Boston, which is a "home," and not a prison with bolts and bars. There is only one restriction, that upon liquor ; its use must be promptly, wholly, and forever abandoned ; in all other respects, there is as much liberty to go and return, to eat, drink, sleep, dress, hunt, fish, and recreate, as a guest would have in the house of a friend. The inmates pay for everything they get, and are as perfectly independent as if they were at an Astor House or a Fifth Avenue. There are libraries and reading-rooms, with newspapers, magazines, and periodicals from all parts of the country; and the associations are as cultivated, scholarly, and mannerly as can be found on board a first-class steamship for the Continent. An ex-patient states that the rules governing the inmates are of the mildest sort. Most excellent attention is given to the sick, and a great variety of rational amusements are provided.

Dr. Parsish, of Philadelphia, presides over another "home," and for his intelligent, self-sacrificing efforts, for years together, to perfect a reform intended to save some of the best hearts and the greatest intellects of the country, he, with those who are engaged in a like humane work, merits and receives the admiration and respect of all good men.

Applicants who are able, pay their own expenses; and often arrangements are made to accommodate those who have no means. In view of all the dreadful calamities which befall a man, sooner or later, who becomes enslaved by drink, let every parent begin early to inspire the minds of children, girls as well as boys, with a terror of the despotism of alcohol; and let it be woven in the frame-work of their minds from day to day, that there is only one way of safety, and that is, never, under any conceivable circumstances, whether of sickness or of pain, to swallow a drop of the accursed thing. Alcohol for the sick is never a necessity; in no case whatever is it more than an inferior substitute or makeshift for something that is much better.

"The most confirmed drunkard cured by a remedy which can be given without the knowledge of the patient" is announced in the daily papers. Should this be true, it will be a great blessing.

